# *Taxillus chinensis* (DC.) Danser: a comprehensive review on botany, traditional uses, phytochemistry, pharmacology, and toxicology

**DOI:** 10.1186/s13020-022-00694-5

**Published:** 2022-12-08

**Authors:** Mi Qin, Qianqian Huang, Xin Yang, Lu Yu, Yong Tang, Chunxiang Zhang, Dalian Qin, Wenjun Zou, Junzhu Deng, Jian Liu, Haiyang Hu, Long Wang, Anguo Wu, Jianming Wu

**Affiliations:** 1 School of Pharmacy, Southwest Medical University, Luzhou, China; 2 Institute of Cardiovascular Research, the Key Laboratory of Medical Electrophysiology, Ministry of Education of China, Medical Key Laboratory for Drug Discovery and Druggability Evaluation of Sichuan Province, Southwest Medical University, Luzhou, China; 3 School of Pharmacy, Chengdu University of Traditional Chinese Medicine, Chengdu, China

**Keywords:** *Taxillus chinensis* (DC.) Danser, Botany, Traditional uses, Phytochemistry, Pharmacology, Toxicology

## Abstract

**Background:**

*Taxillus chinensis* (DC.) Danser (*T. chinensis*), known as “Sangjisheng” in Chinese, is a member of the family *Loranthaceae*, with the traditional functions of “dispelling wind dampness, strengthening bones and muscles, and preventing miscarriage”. Since Eastern Han dynasty, it has been used for the treatment of rheumatoid arthritis, arthralgia, threatened abortion, and hypertension. Nowadays, *T. chinensis* is included in the 2020 Edition of the Chinese Pharmacopoeia as Taxilli Herba. The purpose of this review is to summarize the latest research on *T. chinensis* in recent years, and make critical comments, so as to provide reference for the clinical application and modern research of *T. chinensis*.

**Main body:**

In this review, we summarize the botany, traditional uses, and research advances in the phytochemistry and pharmacological effects of *T. chinensis*. Its toxicity has also been discussed. The published literature on current pharmacological and toxicological data has also been assessed. To date, approximately 110 compounds, including flavonoids, phenolic acids, phenylpropanoids, tannins, glycosides, amino acids, and nucleosides, have been identified in *T. chinensis*. Flavonoids are considered the most vital bioactive ingredients in *T. chinensis*. Pharmacological studies have demonstrated that *T. chinensis* possesses anti-inflammatory, antioxidant, anticancer, antimicrobial, antiviral, diuretic, antihypertensive, antihyperglycemic, and other properties.

**Conclusion:**

Currently, research on *T. chinensis* is in the preliminary stages, and further research is required to understand the active compounds present and mechanisms of action. We hope that this comprehensive review of *T. chinensis* will serve as a background for further research.

## Background

**Fig. 1:**
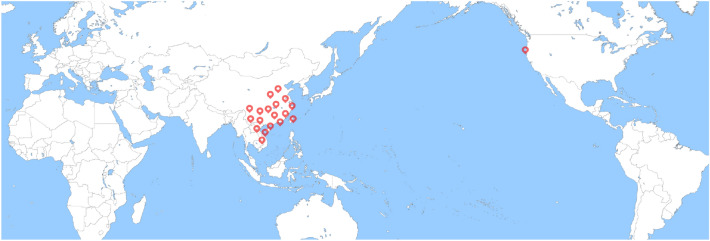
The distribution of *Taxillus chinensis* (DC.) Danser in the world

*T. chinensis* is predominantly found in East Asia, mainly in China, and belongs to the *Loranthaceae* species. *T. chinensis* is commonly found in some alpine and arid desert areas, mainly in southern and southwestern China, such as Guangxi, Fujian, Taiwan, Yunnan, Guangdong, and Hong Kong [1, 2]. In addition, it is distributed in some Southeast Asian nations, such as Malaya, Vietnam, Thailand, Laos, Cambodia, Borneo, and the Philippines (Fig. 1) [3]. *T. chinensis* is a hemi-parasitic plant that is more likely to be parasitic on medium and large trees, such as willow (*Salix babylonica*), maple poplar (*Pterocarya stenoptera*), and platanus (*Platanus acerifolia*). The parasitic position is usually at half the height of the tree or higher.

The chemical composition and pharmacological activity of *T. chinensis* vary depending on its host. In China, Taxilli Herba has been shown to “nourish the liver and kidneys, strengthen tendons and bones, expel wind and dampness, and prevent miscarriages”. It is often used for rheumatism, soreness and weakness of the waist and knees, weakness of muscles and bones, profuse menstrual bleeding, blood leakage during pregnancy, threatened abortion, and high blood pressure. For the past 300 years, people in Guangdong and Guangxi in China have used the stems and buds of *T. chinensis* for making tea. It helps relax muscles and improve collateral circulation. In addition, women in the Lingnan region often use *T. chinensis* to make soups and tea for pregnancy preservation and postpartum rehabilitation. Clinically, it is mainly used for bone and joint diseases, gynecological diseases, and cerebrovascular diseases such as rheumatoid arthritis, arthralgia, miscarriage, hypertension, and obesity [4–8]. Owing to development and improved social awareness regarding healthcare, *T. chinensis* has broad economic prospects in various fields.

To date, approximately 110 chemical constituents have been identified in *T. chinensis,* including flavonoids, phenolic acids, phenylpropanoids, tannins, glycosides, amino acids, and nucleosides. The main chemical constituents in *T. chinensis* are flavonoids, such as quercetin, quercitrin, rutin, avicularin, and small amounts of d-catechol, mainly dihydroflavones, flavones, and flavonolosides, which are the current research hotspots. Many studies have shown that *T. chinensis* exhibits many pharmacological effects, including anti-inflammatory, anticancer, antioxidant, antiviral, antimicrobial, diuretic, antihypertensive, and antihyperglycemic activities. The anti-inflammatory, antioxidant, and hypertensive pharmacological effects are closely related to traditional applications [9]. Primarily, it inhibits the production of inflammatory factors and reduces mitochondrial oxidative stress and cyclin degradation. In addition, *T. chinensis* exhibits both hepatotoxicity and embryotoxicity.

**Fig. 2:**
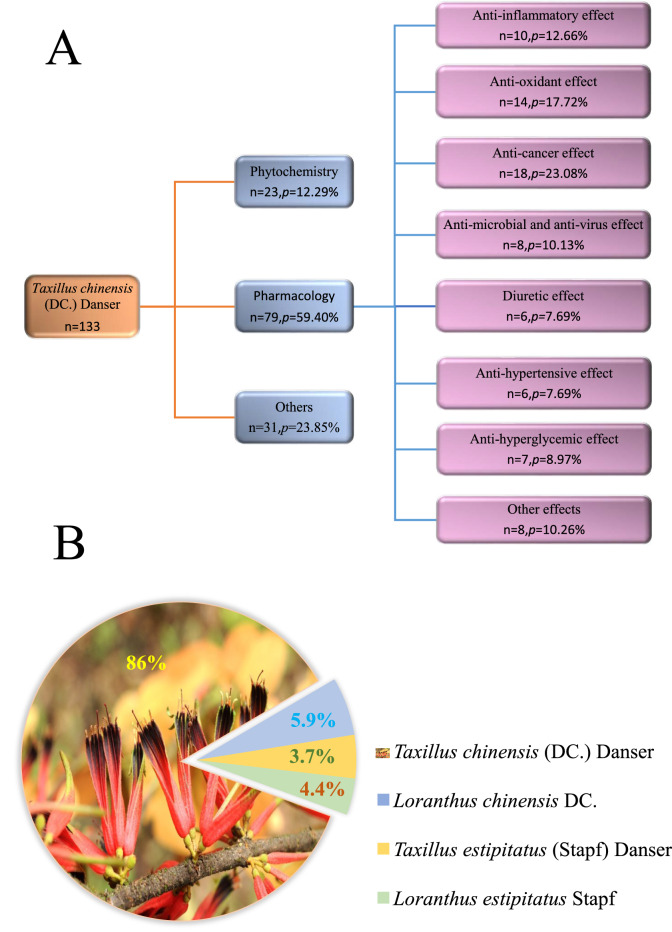
*Taxillus chinensis* (DC.) Danser-related articles cited in this review. **A** The proportion and classification of articles cited in this review. **B** The names commonly used in the literature and data retrieval of *Taxillus chinensis* (DC.) Danser

In this review, relevant studies on *T. chinensis* published before May 2022 were collected from online databases, and more than 360 articles were obtained using the keyword “*Taxillus chinensis* (DC.) Danser” on *Google Scholar*, of which over 70 articles contained the keyword “Sang Ji Sheng.” We analyzed the proportion and classification of the literature cited in this review (Fig. 2A). The names commonly used in the literature and data retrieval for *T. chinensis* are shown in Fig. 2B. At present, the literature on *T. chinensis* is mainly based on the identification of chemical components; however, the components have not been thoroughly analyzed, and only simple pharmacological activity studies have been performed that were mainly focused on crude extracts and their characteristic compounds, especially quercetin. Furthermore, many active components of *T. chinensis* have not yet been fully investigated, nor have their mechanisms of action elucidated. Moreover, bioassessments should consider the advisable effective dose, frequency of administration, and treatment duration. Reports on botany, traditional applications, or toxicology are minimal; therefore, these aspects must be reviewed, in addition to the phytochemistry and pharmacology of *T. chinensis*. This review summarizes the research status, prospects, and limitations of *T. chinensis* and summarizes its relevance to traditional uses, chemical components, and pharmacological activities. It also criticizes the shortcomings of current research. These will provide references for further research, applications, and development.

## Botany

**Fig. 3:**
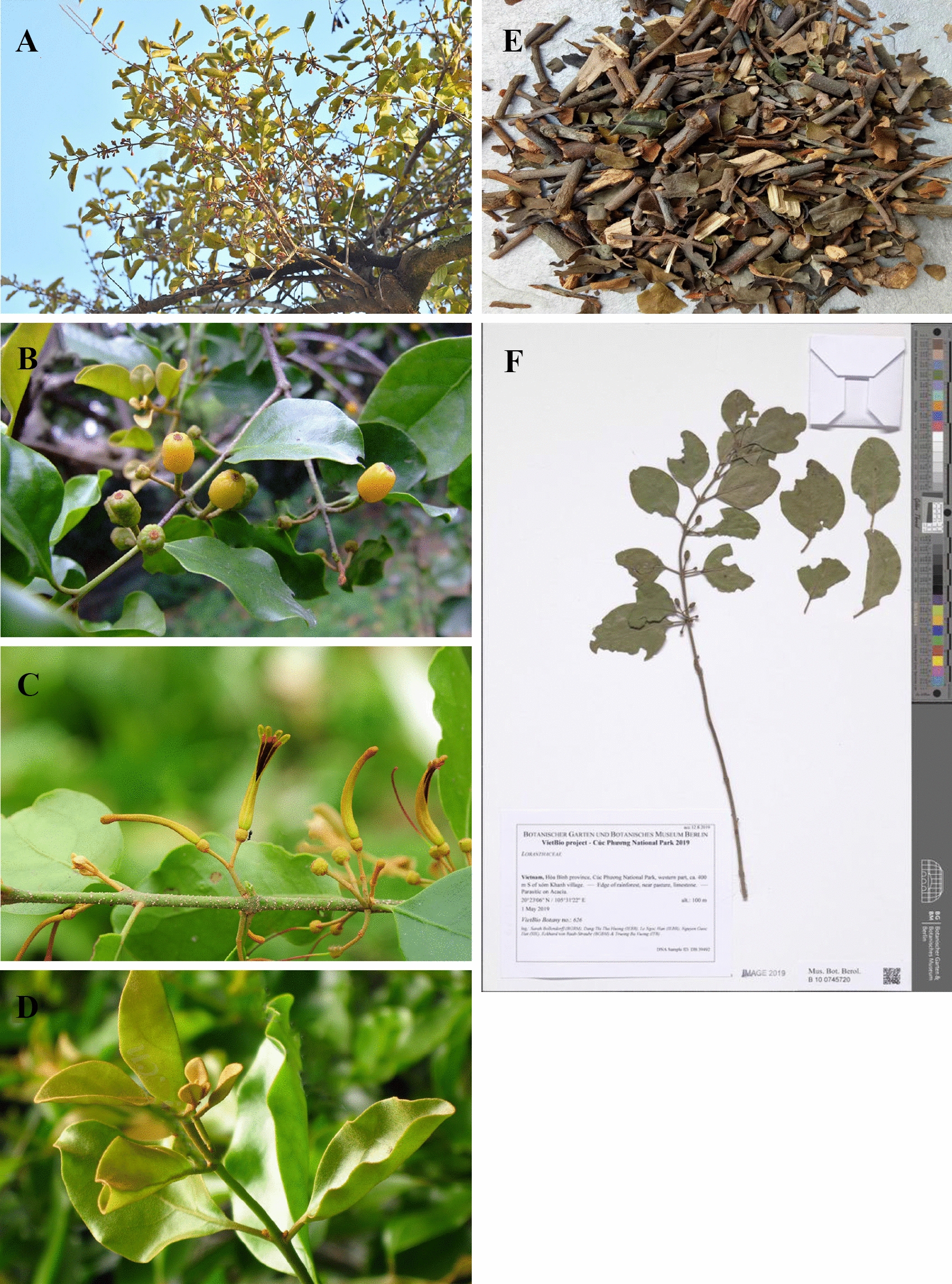
*Taxillus chinensis* (DC.) Danser. (Obtained from http://ppbc.iplant.cn/sp/1295). **A** The whole plant, **B** fruits, **C** flowers, **D** leaves,** E** Taxilli Herba, **F** specimen of *T. chinensis* (https://www.gbif.org/occurrence/3059536395)

According to the Chinese Pharmacopoeia (2020 edition) and “*Zhong Hua Ben Cao*,” *T. chinensis* is a hemi-parasitic shrub that grows to a height of 0.5–1 m [10, 11]. It is usually distributed in plains or low-mountain evergreen broad-leaved forests at altitudes of 20–400 m. Branchlet and leafage: densely covered with brown or reddish-brown stellate hairs, sometimes with scattered or stacked stellate hairs. Branchlets were black and glabrous. Leaves are nearly opposite or alternate, leathery, ovate, long ovate or elliptic, 5–8 cm long, 3–4.5 cm wide, apex obtuse, base suborbicular, glabrous on top, tomentose on the bottom; lateral veins in 4–5 pairs, leaf veins are obvious; petioles 6–12 mm long, glabrous. Flowers: racemes are borne in the leaf axils or leaf axils of twigs, with 2 to 5 flowers, the inflorescence is corymbose, inflorescences and flowers are densely covered with brown stellate hairs; peduncle and rachis 1–3 mm long, pedicels 2–3 mm long; bracts ovate-triangular, 4-toothed; corolla flower bud is tube-shaped, 2.2–2.8 cm long, slightly curved, distend swollen, apically elliptic, lobes 4, lanceolate, 6–9 mm long, reflexed, sparse hair after anthesis; filaments approximately 2 mm long, anthers 3–4 mm long, another room often has a diaphragm, and the stigma is conical. Fruits: elliptical, yellow–green, with granular skin (https://www.plantplus.cn). The entire plant is shown in Fig. 3.

Owing to similarities in morphology and difficulty in sample collection, *T. chinensis* is often confused with other plants, such as *Scurrula parasitica* L., *Taxillus balansae* (Lecomte) Danser, and *Scurrula parastica* var. *graciliflora*. In addition to macroscopic morphological identification, microscopic, physical and chemical, and secondary metabolite analyses, and genetic identification provide more accurate evidence. For example, Wu et al. used ultra-fast liquid chromatography coupled with triple quadrupole mass spectrometry (UFLC-QTRAP-MS/MS) to identify some key constituents, including isoquercitrin, catechin, proline, hyperin, quercetin, etc., to distinguish Taxilli Herba samples from different hosts. The results showed that the relative levels of these components in the *M. alba* sample were higher than those in other samples [12]. The quantitative analysis of these key components is not only important for identifying *T. chinensis* from different host sources, but also plays an important role in the safety and effectiveness of the clinical application of *T. chinensis*. Li et al. evaluated five candidate DNA barcodes, *rbcL*, *matK*, *psbA-trnH*, ITS, and ITS2 DNA, to distinguish *T. chinensis* from adulterants. Among the five barcodes, the amplification and sequencing efficiencies of *rbcL* and *psbA-trnH* were 100%. However, *rbcL* showed no interspecific discrepancy or identification efficiency. In summary, the *psbA-trnH* DNA barcode is of great significance for authentically identifying *T. chinensis* and related parasitic *Loranthus* [13]. Therefore, modern technology plays a significant role in the identification of medicinal materials.

*T. chinensis* contains chlorophyll that is connected to the host through a suction device. Owing to its hemi-parasitic characteristics, in the natural state, the spread and reproduction of *T. chinensis* is mainly achieved by birds pecking at the pulp of *T. chinensis* fruit, discarding seeds, or digesting the fruit to expel the seeds [14].

## Traditional uses

The ancient pharmacological book *“Shennong Bencao Jing*” records *T. chinensis* for the first time, which was listed as “top grade” and included in the 2020 Edition of the Chinese Pharmacopoeia [10, 15]. It is bland in nature, bitter and sweet in flavor, and is attributed to the liver and kidney meridians. The medicinal value was first described in “*Sheng Cao Yao Xing Bei Yao*” (AD1711): “*T. chinensis* can nourish blood and dissipate heat and is used as a tea to relax tendons and activate collaterals” [16]. According to ancient books such as “*Ben Cao Qiu Zhen*” (AD1769), “*Ben Jing Feng Yuan*” (AD1695), and “*Yao Xing Lun*” (Tang Dynasty), *T. chinensis* can “dispel wind, remove dampness, replenish the kidney essence, nourish the liver, strengthen muscles and bones, and prevent miscarriage” [17–19]. Traditionally, it was used to treat painful disabilities caused by wind dampness, pain, and weakness in the lower back and knees, weak tendons and bones, bleeding and spotting, profuse menstruation, vaginal bleeding during pregnancy, threatened abortion, dizziness, and vertigo rheumatism (Table 1). “*Classified Materia Medica*” records: “When you taste the real *T. chinensis* plant, you can experience very good healing effects*.*” Among these, the application of *T. chinensis* in treating threatened abortion is closely related to its function of tonifying the kidney essence and nourishing the liver [20–22].

**Table 1:** Traditional applications of *Taxillus chinensis* (DC.) Danser in classic books of herbal medicine

Traditional uses	Plant parts	Preparations	Usage	Dosage	Classic books
Rheumatism	Branches and leaves	Decoction	Oral administration	15–30 g	*Bei Ji Qian Jin Yao Fang* [121]
Gynecological diseases	Branches and leaves	Pill/decotion	Oral administration	10–15 g	*Yi Xue Zhong Zhong Can Xi Lu; Tai Ping Sheng Hui He Ji Ju Fang* [122, 123]
Backache	Leaves	Tea/dispersant	Oral administration	3–15 g	*Sheng Cao Yao Xing Bei Yao; Ben Cao Gang mu* [16, 124]
Paralysis	Branches and leaves	Pill/decoction	Oral administration	6–30 g	*Shi Yi De Xiao Fang* [125]

Owing to the parasitic characteristics of *Loranthaceae* plants, *T. chinensis* from different hosts is used to treat various diseases in folk medicine. For example, “*Southern Yunnan Materia Medica*” (AD1436) records that *T. chinensis* parasitizing *Styphnolobium japonicum* (L.) Schott can treat intestinal sub-wind blood syndrome, hemorrhoids, and blood leakage; *T. chinensis* parasitizing *Morus alba* L. can treat muscle and collateral obstruction and wind-cold-dampness arthralgia [23]. Its clinical applications must be specified; otherwise, it may be misused, and the desired therapeutic effect will not be achieved. To enhance efficacy, *T. chinensis* is often used in combination with other drugs, such as *Gastrodia* rhizoma, *Smilacis glabrae* Rhizome, *Eucommiae* cortex, and *Polygoni multiflori* Radix. Furthermore, the combined use of *T. chinensis* and *Epimedium brevicornum* Maxim. has a certain effect on the acute stage of poliomyelitis and its sequelae. However, *T. chinensis* may cause disease complications in some patients with liver and heart diseases; further research is required to specify the exact cause for this. In this review, we summarized the commonly used prescriptions containing *T. chinensis*, and their original sources, compositions, traditional effects, and indications are listed in Table 2. The extensive traditional use of *T. chinensis* indicates that it is safe for use in clinical practice.

**Table 2:** The preparations of *Taxillus chinensis* (DC.) Danser in China

Preparation name	Compositions	Traditional effects	Traditional indications	References
Duohuo Jisheng pill (*Bei ji qian jin yao fang*)	*Angelicae sinensis* (Oliv.) Diels, *T. chinensis*, *Rehmanniae glutinosa* (Gaetn.) Libosch. ex Fisch. et Mey., *Achyranthes bidentata* BI., *Asarum sieboldii* Miq., *Gentiana macrophylla*, *Poria cocos* (Schw.) Wolf, *Cinnamomum cassia* Presl, *Saposhnikovia divaricate* (Trucz.) Schishchk., *Ligusticum chunxiong hort*, *Codonopsis Pilosula*, *Glycyrrhiza uralensis* Fisch., *Angelica sinensis* (Oliv.) Diels, *Cynanchum otophyllum* Schneid., *Eucommia Ulmoides*	It has the functions of nourishing blood and relaxing muscles, dispelling wind and dampness, and replenishing liver and kidney	It is used for wind-cold-dampness arthralgia, cold pain in the waist and knees, and unfavorable flexion and extension	[126, 127]
Mi Niao Ning particles (Chinese pharmacopoeia)	*Polygonum aviculare* L., *Phellodendron chinense* Schneid., *Abutilon theophrastii* Medic., *T. chinensis*, *Dipsacus asper* Wall.ex Henry, *Schisandra chinensis* (Turcz.) Baill., *Bupleurum chinense*, *Angelica dahurica* (Fisch. Ex Hoffm.) Benth. Et Hook. f. ex Franch. et Sav, *Glycyrrhiza uralensis* Fisch	It has the functions of clearing heat and dredging, diuretic and pain relief, and invigorating the kidney	It is used for pyretic stranguria, urination redness and pain and urinary tract infections	[128]
Zishen Yutai pill (*Shang han lun*)	*Rehmannia glutinosa* Libosch., *Panax ginseng* C. A. Meyer, *Eucommia ulmoides* Oliver, *Fallopia multiflora* (Thunb.) Harald, *Lycii fructus*, *Asini Corii Colla*, *Cervi cornu* Degelatinatum, *Morinda officinalis* How, *Cuscuta chinensis* Lam., *T. chinensis*, *Dipsacus asper* Wall.ex Henry, *Codonopsis pilosula* (Franch.) Nannf., *Atractylodes macrocephala* Koidz, *Artemisiae argri* Folium, *Amomum villosum* Lour	It has the functions of invigorating the spleen and kidney, reinforcing qi and build up the strong constitution, nourishing blood and preventing abortion, and strengthening the body	It is used for slippery tire caused by deficiency of both spleen and kidney and insufficiency of Chong Ren	[129]
Qisang Yigan pill (*Compilation of national proprietary Chinese medicine*)	*Astragalus membranaceus* (Fisch.) Bunge, *Reynoutria japonica* Houtt., *Sophora flavescens* Ait., *T. chinensis*, *Swertia mileensis*, *Cordyceps sinensis*, *Chinemys reevesii* (Gray), *Panax notoginseng* (Burkill) F. H. Chen ex C. H., mel	It has the functions of invigorating the spleen and kidney, promoting blood circulation and removing blood stasis, clearing dampness and heat	It is used for chronic hepatitis B caused by damp-heat stasis, spleen and kidney deficiency	[130]
Sangge Jiangzhi pill (Chinese pharmacopoeia)	*T. chinensis*, *Pueraria lobata* (Willd.) Ohwi., *Dioscorea opposita* Thunb., *Rhei Radix et Rhizome*, *Crataegus pinnatifida* Bge., *Salvia miltiorrhiza* Bge., *Carthamus tinctorius* L., *Alisma plantago-aquatica* Linn., *Artemisia capillaris* Thunb., *Taraxacum mongolicum* Hand.-Mazz	It has the functions of invigorating the kidney and strengthening the spleen, removing blood stasis, clearing heat and removing dampness	It is used for spleen-kidney deficiency and phlegm turbidity and blood stasis type hyperlipidemia	[131]
Yunkang particles (Chinese pharmacopoeia)	*Dioscorea opposita* Thunb., *Dipsacus asper* Wall. ex Henry, *Astragalus membranaceus*, *Angelica sinensis* (Oliv.) Diels, *Cibotium barometz*, *Cuscuta chinensis*, *T. chinensis*, *Eucommia Ulmoides*, *Psoralea corylifolia* L., *Codonopsis Pilosula*, *Poria cocos*, *Atractylodes macrocephala* Koidz, *Asini Corii Colla*, *Rehmannia glutinosa* Libosch., *Cornus officinalis*, *Lycii fructus*, *Mume fructus*, *Paeonia lactiflora* Pall., *Amomum villosum* Lour., *Alpinia oxyphylla* Miq., *Boehmeria nivea Roots*, *Scutellaria baicalensis* Georgi, *Artemisiae argri* Folium	It has the functions of invigorating the spleen and strengthening the kidney, nourishing the blood and calming the fetus	It is used for threatened abortion and habitual abortion for kidney deficiency type and qi and blood deficiency type	[132, 133]

## Phytochemistry

The metabolic activity of the parasitic plant itself also differs depending on the diversity of the host, which produces different chemical constituents. This review summarizes approximately 110 compounds, including flavonoids, phenolic acids, phenylpropanoids, tannins, glycosides, amino acids, and nucleosides, that have been isolated and identified from *T. chinensis*, mainly *Morus alba*, *Magnifera indica*, and *Liquidambar formosana*. The components of *T. chinensis* are diverse. The chemical constituents and their classifications, structures, chemical formulas, extraction solvents, and parts are shown in Table 3. In the past three years, schools including Nanjing University of Chinese Medicine, Guangzhou University of Chinese Medicine, Chengdu University of Chinese Medicine and Guangxi University of Chinese Medicine have carried out the separation of *T. chinensis*. At present, the separation of *T. chinensis* components is mainly performed using ultrasonic treatment with different concentrations of methanol. In order to separate more active ingredients, more treatment methods and extraction solvents may be tried in subsequent work.

**Table 3:** Chemical compounds isolated from *Taxillus chinensis* (DC.) Danser

No	Chemical components	Classification	Structure	Formula	Extraction solvent and parts	References
	Flavonoids					
** 1**	Isosakuranetin	Dihydroflavones		C_16_H_14_O_5_	50% methanol extract of the dried stems and branches with leaves of *T. chinensis* from *Morus alba* L	[27]
** 2**	Prunin	Dihydroflavones		C_21_H_22_O_10_	50% methanol extract of the dried stems and branches with leaves of *T. chinensis* from *Morus alba* L	[27]
** 3**	Taxifolin	Dihydroflavonols		C_15_H_12_O_7_	50% methanol extract of the dried stems and branches with leaves of *T. chinensis* from *Morus alba* L	[27]
** 4**	3'-*O*-methyl-dihydroquercetin-7-*β-*D-glucoside	Dihydroflavonols		C_22_H_24_O_12_	50% methanol extract of the dried stems and branches with leaves of *T. chinensis* from *Morus alba* L	[27]
** 5**	Taxifolin 3'-*O*-*β*-D-glucopyranoside	Dihydroflavonols		C_21_H_22_O_12_	50% methanol extract of the dried stems and branches with leaves of *T. chinensis* from *Morus alba* L	[27]
** 6**	Quercetin	Flavonols		C_15_H_10_O_7_	50% methanol extract of the dried stems and branches with leaves of *T. chinensis* from *Morus alba* L	[27]
** 7**	7-*O*-benzyl luteolin	Flavones		C_22_H_16_O_6_	50% methanol extract of the dried stems and branches with leaves of *T. chinensis* from *Morus alba* L	[27]
** 8**	Cosmosiin	Flavones		C_21_H_20_O_10_	50% methanol extract of the dried stems and branches with leaves of *T. chinensis* from *Morus alba* L	[27]
** 9**	Luteolin-7-*O*-glucoside	Flavones		C_21_H_20_O_11_	50% methanol extract of the dried stems and branches with leaves of *T. chinensis* from *Morus alba* L	[27]
** 10**	Tricetin	Flavones		C_15_H_10_O_7_	50% methanol extract of the dried stems and branches with leaves of *T. chinensis* from *Morus alba* L	[27]
** 11**	5-demethylnobiletin	Flavones		C_20_H_20_O_8_	50% methanol extract of the dried stems and branches with leaves of *T. chinensis* from *Morus alba* L	[27]
** 12**	(+)-catechin	Flavanes		C_15_H_14_O_6_	50% methanol extract of the dried stems and branches with leaves of *T. chinensis* from *Morus alba* L	[27]
** 13**	Epicatechin	Flavanes		C_15_H_14_O_6_	50% methanol extract of the dried stems and branches with leaves of *T. chinensis* from *Morus alba* L	[27]
** 14**	Epigallocatechin	Flavanes		C_15_H_14_O_7_	50% methanol extract of the dried stems and branches with leaves of *T. chinensis* from *Morus alba* L	[27]
** 15**	(−)-epicatechin gallate	Flavanes		C_22_H_18_O_10_	50% methanol extract of the dried stems and branches with leaves of *T. chinensis* from *Morus alba* L	[27]
** 16**	Kaempferitrin	Flavonolosides		C_27_H_30_O_14_	50% methanol extract of the dried stems and branches with leaves of *T. chinensis* from *Morus alba* L	[27]
** 17**	Brassicin	Flavonolosides		C_22_H_22_O_12_	50% methanol extract of the dried stems and branches with leaves of *T. chinensis* from *Morus alba* L	[27]
** 18**	Galangin	Flavonolosides		C_15_H_10_O_5_	50% methanol extract of the dried stems and branches with leaves of *T. chinensis* from *Morus alba* L	[27]
** 19**	Quercetin-3-*O*-(6''- galloyl)-*β*- galactopyranside	Flavonolosides		C_28_H_24_O_16_	50% methanol extract of the dried stems and branches with leaves of *T. chinensis* from *Morus alba* L	[27]
** 20**	Quercetin-3-*O*-(6''-galloyl)-*β*-D-glucopyranoside	Flavonolosides		C_28_H_24_O_16_	70% methanol extract of the stems, branches and leaves of *T. chinensis* from 45 hosts	[12]
** 21**	Myricetrin	Flavonolosides		C_21_H_20_O_12_	50% methanol extract of the dried stems and branches with leaves of *T. chinensis* from *Morus alba* L	[27]
** 22**	Rutin	Flavonolosides		C_27_H_30_O_16_	50% methanol extract of the dried stems and branches with leaves of *T. chinensis* from *Morus alba* L	[27]
** 23**	Hyperoside	Flavonolosides		C_21_H_20_O_12_	50% methanol extract of the dried stems and branches with leaves of *T. chinensis* from *Morus alba* L	[27]
** 24**	Quercetin 3-*O-β-*D- glucuronide	Flavonolosides		C_21_H_18_O_13_	50% methanol extract of the dried stems and branches with leaves of *T. chinensis* from *Morus alba* L	[27]
** 25**	Isoquercitrin	Flavonolosides		C_21_H_20_O_12_	50% methanol extract of the dried stems and branches with leaves of *T. chinensis* from *Morus alba* L	[27]
** 26**	Avicularin	Flavonolosides		C_20_H_18_O_11_	50% methanol extract of the dried stems and branches with leaves of *T. chinensis* from *Morus alba* L	[27]
** 27**	Kaempferol-3-rutinoside	Flavonolosides		C_27_H_30_O_15_	50% methanol extract of the dried stems and branches with leaves of *T. chinensis* from *Morus alba* L	[27]
** 28**	Quercitrin	Flavonolosides		C_21_H_20_O_11_	50% methanol extract of the dried stems and branches with leaves of *T. chinensis* from *Morus alba* L	[27]
**29**	Astragalin	Flavonolosides		C_21_H_20_O_11_	50% methanol extract of the dried stems and branches with leaves of *T. chinensis* from *Morus alba* L	[27]
** 30**	Afzelin	Flavonolosides		C_21_H_20_O_10_	50% methanol extract of the dried stems and branches with leaves of *T. chinensis* from *Morus alba* L	[27]
** 31**	Homomangiferin	Bisphenirone flavonoids		C_20_H_20_O_11_	50% methanol extract of the dried stems and branches with leaves of *T. chinensis* from *Morus alba* L	[27]
** 32**	Isochinomin	Bisphenirone flavonoids		C_20_H_20_O_11_	50% methanol extract of the dried stems and branches with leaves of *T. chinensis* from *Morus alba* L	[27]
** 33**	Hydrocarpin	Flavonoid lignans		C_25_H_20_O_9_	50% methanol extract of the dried stems and branches with leaves of *T. chinensis* from *Morus alba* L	[27]
** 34**	8-methylretusin-7-*β*- glucoside	Isoflavones		C_23_H_24_O_10_	50% methanol extract of the dried stems and branches with leaves of *T. chinensis* from *Morus alba* L	[27]
** 35**	Cynaroside A	Flavonoids		C_21_H_32_O_10_	50% methanol extract of the dried stems and branches with leaves of *T. chinensis* from *Morus alba* L	[27]
** 36**	Luteolin-7,3'-di-*O*-*β*-D-glucoside	Flavonoids	not found	C_27_H_30_O_16_	70% methanol extract of the stems and leaves of *T. chinensis* from *Morus alba* L	[28]
** 37**	Pinocembrin	Flavonoids		C_15_H_12_O_4_	70% methanol extract of the stems and leaves of *T. chinensis* from *Morus alba* L	[28]
** 38**	Nigrasins A	Flavonoids		C_25_H_26_O_8_	70% methanol extract of the stems and leaves of *T. chinensis* from *Morus alba* L	[28]
** 39**	Artonin E2	Flavonoids		C_25_H_24_O_7_	70% methanol extract of the stems and leaves of *T. chinensis* from *Morus alba* L	[28]
** 40**	Luteolin 8-C-hexosyl-*O*-hexoside	Flavonoids	not found	C_27_H_30_O_16_	70% methanol extract of the stems and leaves of *T. chinensis* from *Morus alba* L	[28]
** 41**	Sanggenon F/H	Flavonoids		C_20_H_18_O_6_	70% methanol extract of the stems and leaves of *T. chinensis* from *Morus alba* L	[28]
** 42**	Kuwanon S2	Flavonoids		C_25_H_26_O_5_	70% methanol extract of the stems and leaves of *T. chinensis* from *Morus alba* L	[28]
** 43**	Sanggenon M	Flavonoids		C_25_H_24_O_7_	70% methanol extract of the stems and leaves of *T. chinensis* from *Morus alba* L	[28]
** 44**	Kuwanon D	Flavonoids		C_25_H_26_O_6_	70% methanol extract of the stems and leaves of *T. chinensis* from *Morus alba* L	[28]
** 45**	Sanggenol L	Flavonoids		C_25_H_26_O_6_	70% methanol extract of the stems and leaves of *T. chinensis* from *Morus alba* L	[28]
** 46**	Mangiferin	Flavonoids		C_19_H_18_O_11_	40% methanol extract of the branches and leaves of *T. chinensis* from *Mangifera indica*	[29]
** 47**	Quercetin-3-*O*-*β*-D-glucuronide	Flavonoids		C_21_H_18_O_13_	70% methanol extract of the stems, branches and leaves of *T. chinensis* from 45 hosts	[12]
** 48**	Hyperin	Flavonoids		C_21_H_20_O_12_	70% methanol extract of the stems, branches and leaves of *T. chinensis* from 45 hosts	[12]
** 49**	Kaempferol-3,7- bisrhamnoside	Flavonoids		C_27_H_30_O_14_	70% methanol extract of the stems, branches and leaves of *T. chinensis* from 45 hosts	[12]
	Phenolic acids					
** 50**	Quinic acid	Phenolic acids		C_7_H_12_O_6_	50% methanol extract of the dried stems and branches with leaves of *T. chinensis* from *Morus alba* L	[27]
** 51**	Shikimic acid	Phenolic acids		C_7_H_10_O_5_	50% methanol extract of the dried stems and branches with leaves of *T. chinensis* from *Morus alba* L	[27]
** 52**	Malic acid	Phenolic acids		C_4_H_6_O_5_	50% methanol extract of the dried stems and branches with leaves of *T. chinensis* from *Morus alba* L	[27]
** 53**	Citric acid	Phenolic acids		C_6_H_8_O_7_	50% methanol extract of the dried stems and branches with leaves of *T. chinensis* from *Morus alba* L	[27]
** 54**	Gallic acid	Phenolic acids		C_7_H_6_O_5_	50% methanol extract of the dried stems and branches with leaves of *T. chinensis* from *Morus alba* L	[27]
** 55**	Protocatechuic acid	Phenolic acids		C_7_H_6_O_4_	50% methanol extract of the dried stems and branches with leaves of *T. chinensis* from *Morus alba* L	[27]
** 56**	4-hydroxybenzoic acid	Phenolic acids		C_7_H_6_O_3_	50% methanol extract of the dried stems and branches with leaves of *T. chinensis* from *Morus alba* L	[27]
** 57**	Coniferic acid	Phenolic acids		C_10_H_10_O_4_	70% methanol extract of the stems, branches and leaves of *T. chinensis* from 45 hosts	[12]
	Phenylpropanoids					
** 58**	Toddacoumaquinone	Phenylpropanoids		C_23_H_18_O_7_	50% methanol extract of the dried stems and branches with leaves of *T. chinensis* from *Morus alba* L	[27]
** 59**	p-coumaric acid	Phenylpropanoids		C_9_H_8_O_3_	50% methanol extract of the dried stems and branches with leaves of *T. chinensis* from *Morus alba* L	[27]
** 60**	Caffeic acid	Phenylpropanoids		C_9_H_8_O_4_	50% methanol extract of the dried stems and branches with leaves of *T. chinensis* from *Morus alba* L	[27]
** 61**	Chlorogenic acid	Phenylpropanoids		C_16_H_18_O_9_	50% methanol extract of the dried stems and branches with leaves of *T. chinensis* from *Morus alba* L	[27]
	Tannins					
** 62**	Glucogallin	Hydrolysable tannins		C_13_H_16_O_10_	50% methanol extract of the dried stems and branches with leaves of *T. chinensis* from *Morus alba* L	[27]
** 63**	Procyanidin B2	Condensed tannins		C_30_H_26_O_12_	50% methanol extract of the dried stems and branches with leaves of *T. chinensis* from *Morus alba* L	[27]
** 64**	Procyanidin B1	Condensed tannins		C_30_H_26_O_12_	50% methanol extract of the dried stems and branches with leaves of *T. chinensis* from *Morus alba* L	[27]
** 65**	Procyanidin C1	Condensed tannins		C_45_H_38_O_18_	50% methanol extract of the dried stems and branches with leaves of *T. chinensis* from *Morus alba* L	[27]
** 66**	Procyanidin B2 3'-*O*-gallate	Condensed tannins		C_37_H_30_O_16_	50% methanol extract of the dried stems and branches with leaves of *T. chinensis* from *Morus alba* L	[27]
	Glycosides					
** 67**	Taxilluside A	Ester glycosides		C_18_H_24_O_10_	60% aqueous ethanol extract of the branches and leaves of *T. chinensis*	[40]
** 68**	Taxilluside B	Ester glycosides		C_18_H_24_O_10_	60% aqueous ethanol extract of the branches and leaves of *T. chinensis*	[40]
** 69**	Taxilluside C	Ester glycosides		C_25_H_28_O_14_	60% aqueous ethanol extract of the branches and leaves of *T. chinensis*	[40]
** 70**	Taxilluside D	Ester glycosides		C_18_H_25_O_10_	60% aqueous ethanol extract of the branches and leaves of *T. chinensis*	[40]
** 71**	Hydroxybenzoic acid *β*-D-glucose ester	Ester glycosides		C_13_H_16_O_8_	50% methanol extract of the dried stems and branches with leaves of *T. chinensis* from *Morus alba* L	[27]
** 72**	Tachioside	Phenol glycosides		C_13_H_18_O_8_	50% methanol extract of the dried stems and branches with leaves of *T. chinensis* from *Morus alba* L	[27]
** 73**	(1S)-2(acetyloxy)-1-(hydroxymethy)ethyl-*β*-D-glucopyranoside	Phenol glycosides		C_11_H_20_O_9_	50% methanol extract of the dried stems and branches with leaves of *T. chinensis* from *Morus alba* L	[27]
** 74**	Gallic acid 3-*O-β-*D-glucopyranoside	Phenol glycosides		C_13_H_16_O_10_	50% methanol extract of the dried stems and branches with leaves of *T. chinensis* from *Morus alba* L	[27]
** 75**	2,6-dihydroxyphenyl-*β-*D-glucopyranosiduronic acid	Phenol glycosides		C_12_H_14_O_9_	50% methanol extract of the dried stems and branches with leaves of *T. chinensis* from *Morus alba* L	[27]
** 76**	Glucosyringic acid	Phenol glycosides		C_15_H_20_O_10_	50% methanol extract of the dried stems and branches with leaves of *T. chinensis* from *Morus alba* L	[27]
** 77**	Methyl-4-(*β-*D*-*glucopyranosyloxy)-3-methoxybenzoate	Phenol glycosides		C_15_H_20_O_9_	50% methanol extract of the dried stems and branches with leaves of *T. chinensis* from *Morus alba* L	[27]
** 78**	Ethyl 3-(*β*-D-glucopyranosyloxy) butanoate	Phenol glycosides		C_12_H_22_O_8_	50% methanol extract of the dried stems and branches with leaves of *T. chinensis* from *Morus alba* L	[27]
** 79**	Hexyl 6-*O-β-*D-xylopyranosyl-*β-*D-glucopyranoside	Phenol glycosides		C_17_H_32_O_10_	50% methanol extract of the dried stems and branches with leaves of *T. chinensis* from *Morus alba* L	[27]
** 80**	Diyhdromelilotoside	Phenol glycosides		C_15_H_20_O_8_	50% methanol extract of the dried stems and branches with leaves of *T. chinensis* from *Morus alba* L	[27]
** 81**	Androsin	Phenol glycosides		C_15_H_20_O_8_	50% methanol extract of the dried stems and branches with leaves of *T. chinensis* from *Morus alba* L	[27]
** 82**	Phloracetophenone 4'-*O-*glucoside	Phenol glycosides		C_14_H_18_O_9_	50% methanol extract of the dried stems and branches with leaves of *T. chinensis* from *Morus alba* L	[27]
** 83**	Salicin	Phenol glycosides		C_13_H_18_O_7_	methanol extract of the branches and leaves of *T. chinensis* from Willow	[41]
** 84**	Mulberroside F	Stibene glucosides		C_26_H_30_O_14_	50% methanol extract of the dried stems and branches with leaves of *T. chinensis* from *Morus alba* L	[27]
	Amino acids					
** 85**	Glutamine	Amino acids		C_5_H_10_N_2_O_3_	50% methanol extract of the dried stems and branches with leaves of *T. chinensis* from *Morus alba* L	[27]
** 86**	Lysine	Amino acids		C_6_H_14_N_2_O_2_	70% methanol extract of the stems, branches and leaves of *T. chinensis* from 45 hosts	[12]
** 87**	Histidine	Amino acids		C_6_H_9_N_3_O_2_	70% methanol extract of the stems, branches and leaves of *T. chinensis* from 45 hosts	[12]
** 88**	Argnine	Amino acids		C_6_H_14_N_4_O_2_	70% methanol extract of the stems, branches and leaves of *T. chinensis* from 45 hosts	[12]
** 89**	Serine	Amino acids		C_3_H_7_NO_3_	70% methanol extract of the stems, branches and leaves of *T. chinensis* from 45 hosts	[12]
** 90**	Threonine	Amino acids		C_4_H_9_NO_3_	70% methanol extract of the stems, branches and leaves of *T. chinensis* from 45 hosts	[12]
** 91**	Glutamic acid	Amino acids		C_5_H_9_NO_4_	70% methanol extract of the stems, branches and leaves of *T. chinensis* from 45 hosts	[12]
** 92**	Proline	Amino acids		C_5_H_9_NO_2_	70% methanol extract of the stems, branches and leaves of *T. chinensis* from 45 hosts	[12]
** 93**	Valine	Amino acids		C_5_H_11_NO_2_	70% methanol extract of the stems, branches and leaves of *T. chinensis* from 45 hosts	[12]
** 94**	Tyrosine	Amino acids		C_9_H_11_NO_3_	70% methanol extract of the stems, branches and leaves of *T. chinensis* from 45 hosts	[12]
** 95**	Isoleucine	Amino acids		C_6_H_13_NO_2_	70% methanol extract of the stems, branches and leaves of *T. chinensis* from 45 hosts	[12]
** 96**	Leucine	Amino acids		C_6_H_13_NO_2_	70% methanol extract of the stems, branches and leaves of *T. chinensis* from 45 hosts	[12]
** 97**	Phenylalanine	Amino acids		C_9_H_11_NO_2_	70% methanol extract of the stems, branches and leaves of *T. chinensis* from 45 hosts	[12]
	Nucleotides					
** 98**	2'-deoxyadenosine	Nucleotides		C_10_H_13_N_5_O_3_	70% methanol extract of the stems, branches and leaves of *T. chinensis* from 45 hosts	[12]
** 99**	Inosine	Nucleotides		C_10_H_12_N_4_O_5_	70% methanol extract of the stems, branches and leaves of *T. chinensis* from 45 hosts	[12]
** 100**	Guanosine	Nucleotides		C_10_H_13_N_5_O_5_	70% methanol extract of the stems, branches and leaves of *T. chinensis* from 45 hosts	[12]
** 101**	2'-deoxyguanosine	Nucleotides		C_10_H_13_N_5_O_4_	70% methanol extract of the stems, branches and leaves of *T. chinensis* from 45 hosts	[12]
** 102**	Adenosine	Nucleotides		C_10_H_13_N_5_O_4_	70% methanol extract of the stems, branches and leaves of *T. chinensis* from 45 hosts	[12]
	Others					
** 103**	Glucose	Polyhydroxy aldehydes		C_6_H_12_O_6_	50% methanol extract of the dried stems and branches with leaves of *T. chinensis* from *Morus alba* L	[27]
** 104**	Primeverose	Carbohydrates		C_11_H_20_O_10_	50% methanol extract of the dried stems and branches with leaves of *T. chinensis* from *Morus alba* L	[27]
** 105**	Bisdemethoxycurcumin	Diphenylheptane		C_19_H_16_O_4_	50% methanol extract of the dried stems and branches with leaves of *T. chinensis* from *Morus alba* L	[27]
** 106**	Fluorescein-*β-*D-galactopyranoside	Galactosides		C_26_H_22_O_10_	50% methanol extract of the dried stems and branches with leaves of *T. chinensis* from *Morus alba* L	[27]
** 107**	Artonol B	Phenols		C_24_H_20_O_7_	50% methanol extract of the dried stems and branches with leaves of *T. chinensis* from *Morus alba* L	[27]
** 108**	Swertiamarin	Liridoid		C_16_H_22_O_10_	50% methanol extract of the dried stems and branches with leaves of *T. chinensis* from *Morus alba* L	[27]
** 109**	Emodin	Anthraquinones		C_15_H_10_O_5_	50% methanol extract of the dried stems and branches with leaves of *T. chinensis* from *Morus alba* L	[27]

In addition, researchers have determined the chemical components and their variation laws with significant differences through core means, such as similarity evaluation, cluster analysis, and principal component analysis. Upon analysing these studies, we found that the chemical compositions of *T. chinensis* samples from different hosts were roughly similar, but were clearly different in the contents of flavonoids. Yuan et al. identified 19 different chemical components, including proanthocyanidin B2, proanthocyanidin B1, proanthocyanidin C1, isosakuranetin, and kaempferitrin, in *T. chinensis* from 10 host plants. Among them, ( +)-catechin, hyperoside, quercetin 3-*O*-*β*-D-glucuronide, and quercetin were common chemical components. These four compounds were all flavonoids, but their content differed significantly in *T. chinensis* from different hosts. The relative contents of these four compounds in *T. chinensis* from *Morus alba* L. were higher than those in the other nine hosts, followed by *Clausena lansium* (Lour.) skeels and *Diospyros kaki* Thunb. The quercetin 3-*O*-*β*-D-glucuronide and hyperoside contents were the lowest in *T. chinensis* from *Sapindus saponaria* Linnaeus [24, 25].

## Flavonoids

Flavonoids are widespread throughout the plant kingdom and are considered the main chemical constituents of *T. chinensis* [26]. To date, 49 flavonoids have been identified in *T. chinensis*, including dihydroflavones, flavonols, dihydroflavonols, isoflavones, flavones, and flavanes. A study conducted on a 50% methanol extract of Taxilli Herba from *Morus alba* L. has reported the isolation of 34 flavonoid compounds including isosakuranetin (**1**), prunin (**2**), taxifolin (**3**), 3'-*O*-methyl-dihydroquercetin-7-*β-*D-glucoside (**4**), taxifolin 3'-*O*-*β*-D-glucopyranoside (**5**), quercetin (**6**), 7-*O*-benzyl luteolin (**7**), cosmosiin (**8**), luteolin-7-*O*-glucoside (**9**), tricetin (**10**), 5-demethylnobiletin (**11**), ( +)-catechin (**12**), epicatechin (**13**), epigallocatechin (**14**), (-)-epicatechin gallate (**15**), kaempferitrin (**16**), brassicin (**17**), galangin (**18**), quercetin-3-*O*-(6''-galloyl)-*β*-galactopyranside (**19**), myricetrin (**21**), rutin (**22**), hyperoside (**23**), quercetin 3-*O-β-*D-glucuronide (**24**), isoquercitrin (**25**), avicularin (**26**), kaempferol-3-rutinoside (**27**), quercitrin (**28**), astragalin (**29**), afzelin (**30**), homomangiferin (**31**), isochinomin (**32**), hydrocarpin (**33**), 8-methylretusin-7-*β*-glucoside (**34**), and cynaroside A (**35**) [27]. The 70% methanol extract of Taxilli Herba from 45 hosts by UFLC-QTRAP-MS/MS revealed the presence of flavonoids such as quercetin-3-*O*-(6''-galloyl)-*β*-D-glucopyranoside (**20**), quercetin-3-*O*-*β*-D-glucuronide (**47**), hyperin (**48**), and kaempferol-3,7-bisrhamnoside (**49**) [12]. Ten flavonoid compounds reported using UPLC-MS analysis of the 70% methanol extract of the stems and leaves of *T. chinensis* from *Morus alba* L. were luteolin-7,3'-di-*O*-*β*-D-glucoside (**36**), pinocembrin (**37**), nigrasins A (**38**), artonin E2 (**39**), luteolin 8-C-hexosyl-*O*-hexoside (**40**), sanggenon F/H (**41**), kuwanon S2 (**42**), sanggenon M (**43**), kuwanon D (**44**), and sanggenol L (**45**) [28]. Similarly, mangiferin was found in a 40% methanol extract of branches and leaves of *T. chinensis* from *Mangifera indica* revealed the presence of mangiferin (**46**) [29]. Different extraction sites can have different effects on the content of the active ingredients. The total flavonoid content in the leaves of *T. chinensis* was approximately four times higher than that in the stems and branches [30]. Flavonoids have anti-inflammatory properties and are potent antioxidants [31]. Quercetin 3-*O*-*β*-D-glucuronide, isoquercitrin, catechin, hyperin, hyperoside, quercetin, avicularin, and quercitrin are the main flavonoids in *T. chinensis* and may be the active ingredients in *T. chinensis* that exert anti-inflammatory and antioxidant effects. Of these, quercetin is often sold as a dietary supplement. In Canada, quercetin can be used as a “natural health product” with two different benefits: “antioxidant” or “used in herbal medicine as a capillary or blood vessel protectant” [32]. Rutin is a unique antioxidant flavonoid that is abundant in plants [33]. Isoquercitrin has a variety of protective roles in vitro and in vivo, particularly against oxidative stress, cancer, cardiovascular disease, diabetes, and allergic reactions [34]. Although flavonoids are the hot spots of current research, their structure–activity relationship, activity mechanism, absorption, and metabolism mechanism remain to be understood, which limits their development and utilization. In the future, these aspects of flavonoids should be further studied, as they will have a significant impact on the development of new drugs.

## Phenolic acids

The term “phenolic acids” generally refers to phenols with a single carboxylic acid functionality, and can be categorized into three main subclasses, including hydroxybenzoic acid, hydroxycinnamic acid, and hydroxyphenylacetic acid [35]. At present, 8 phenolic acids have been found in Taxilli Herba, including quinic acid (**50**), shikimic acid (**51**), malic acid (**52**), citric acid (**53**), gallic acid (**54**), protocatechuic acid (**55**), 4-hydroxybenzoic acid (**56**), and coniferic acid (**57**) [12, 27]. Phenolic acids are receiving increasing attention because of their high antioxidant activity and other antibacterial, dietary, and health benefits [36]. However, the proportion of phenolic acids in *T. chinensis* remains unclear; therefore, their biological activity requires further verification. Plant phenolic acids also face problems of low extraction efficiency and low purity, and extraction technology must be continuously improved.

## Phenylpropanoids

Phenylpropanoids are usually a type C6-C3 compound as the basic unit, typically including simple phenylpropanoids, coumarins, and lignans. To date, four phenylpropanoids have been isolated from a 50% methanol extract of Taxilli Herba, including p-coumaric acid (**59**), caffeic acid (**60**), and chlorogenic acid (**61**), which are classified as simple phenylpropanoids, and toddacoumaquinone (**58**), which is a coumarin [27]. Among them, caffeic acid and chlorogenic acid have broad-spectrum antibacterial and antiviral effects, and may be effective components of *T. chinensis*. In the future, the phenylpropane components of *T. chinensis* must be analyzed quantitively and its antiviral activity verified.

## Tannins

A study conducted on a 50% methanol extract of Taxilli Herba from *Morus alba* L. revealed the presence of five tannins, including glucogallin (**62**), procyanidin B2 (**63**), procyanidin B1 (**64**), procyanidin C1 (**65**), and procyanidin B2-3'-*O*-gallate (**66**) [27]. Among them, glucogallin is a hydrolyzable tannin, and the remainder is condensed tannins. Procyanidins are the main components of *T. chinensis* tannins and have various pharmacological activities, including antioxidant, anticancer, and hyperglycemic activities [37–39]. According to current experimental research, they may be the active ingredients responsible for the anticancer activity of *T. chinensis*.

## Glycosides

Glycosides are compounds in which sugars or glycoside saccharides are attached to form another non-sugar substance by carbon atoms at the end of the sugar. To date, 18 glycosides have been isolated and identified from *T. chinensis*. Phytochemical research on the branches and leaves of *T. chinensis* led to the isolation of taxilluside A-D (**67–70**) [40]. Similarly, a study conducted on a 50% methanol extract of Taxilli Herba from *Morus alba* L. reported the isolation of 13 glycoside compounds, including hydroxybenzoic acid *β*-D-glucose ester (**71**), tachioside (**72**), (1S)-2(acetyloxy)-1-(hydroxymethy)ethyl-*β-*D-glucopyranoside (**73**), gallic acid 3-*O-β*-D-glucopyranoside (**74**), 2,6-dihydroxyphenyl-*β*-D-glucopyranosiduronic acid (**75**), glucosyringic acid (**76**), methyl-4-(*β*-D*-*glucopyranosyloxy)-3-methoxybenzoate (**77**), ethyl 3-(*β*-D-glucopyranosyloxy) butanoate (**78**), hexyl 6-*O*-*β*-D-xylopyranosyl-*β-*D-glucopyranoside (**79**), diyhdromelilotoside (**80**), androsin (**81**), phloracetophenone 4'-*O-*glucoside (**82**), and mulberroside F (**84**) [27]. Additionally, salicin (**83**) was isolated from the methanol extract of the branches and leaves of *T. chinensis* in willow [41]. Taxilluside C and taxilluside D from *T. chinensis* exhibited inhibitory activity against calcium concentration in myocardial cells, which was observed by measuring the change in fluorescence signal using laser scanning confocal microscopy [40]. This suggests that *T. chinensis* might have a protective effect on cardiomyocytes. Furthermore, the ion channel regulatory activities of these compounds must be explored further. The activity of other glycosides in *T. chinensis* have not been studied, and will be a future research direction.

## Amino acids

Amino acids are not only used in protein biosynthesis, but also serve as the building blocks of several other biosynthetic pathways and play key roles in signaling processes and plant stress responses [42]. To date, only 13 amino acids have been identified in *T. chinensis*. Glutamine (**85**) was detected in a 50% methanol extract of Taxilli Herba from *Morus alba* L. [27]. A UFLC-QTRAP-MS/MS study conducted on a 70% methanol extract of Taxilli Herba from 45 hosts reported 12 amino acids, including lysine (**86**), histidine (**87**), arginine (**88**), serine (**89**), threonine (**90**), glutamic acid (**91**), proline (**92**), valine (**93**), tyrosine (**94**), isoleucine (**95**), leucine (**96**), and phenylalanine (**97**) [12]. Glutamic acid, arginine, glutamine, and other amino acids can be used alone for treating certain diseases, such as liver, digestive tract, and respiratory diseases [43–45]. Hence, the discovery of amino acids in *T. chinensis* may lead to further research on its potential pharmacological activities.

## Nucleotides

Nucleotides fulfill many essential functions in plants, such as promoting growth, development, and delaying senescence. Five nucleotides, namely 2'-deoxyadenosine (**98**), inosine (**99**), guanosine (**100**), 2'-deoxyguanosine (**101**), and adenosine (**102**), were isolated from a 70% methanol extract of Taxilli Herba from 45 hosts [12]. The nucleotide content of *T. chinensis* plants is unknown. Primarily, it regulates plant growth and development and has little value in terms of pharmacological activity.

## Others

Several other compounds have been isolated from the 50% methanol extract of Taxilli Herba from *Morus alba* L., such as glucose (**103**), primeverose (**104**), bisdemethoxycurcumin (**105**), fluorescein-*β-*D-galactopyranoside (**106**), artonol B (**107**), swertiamarin (**108**), and emodin (**109**) [27].

## Pharmacology

**Fig. 4:**
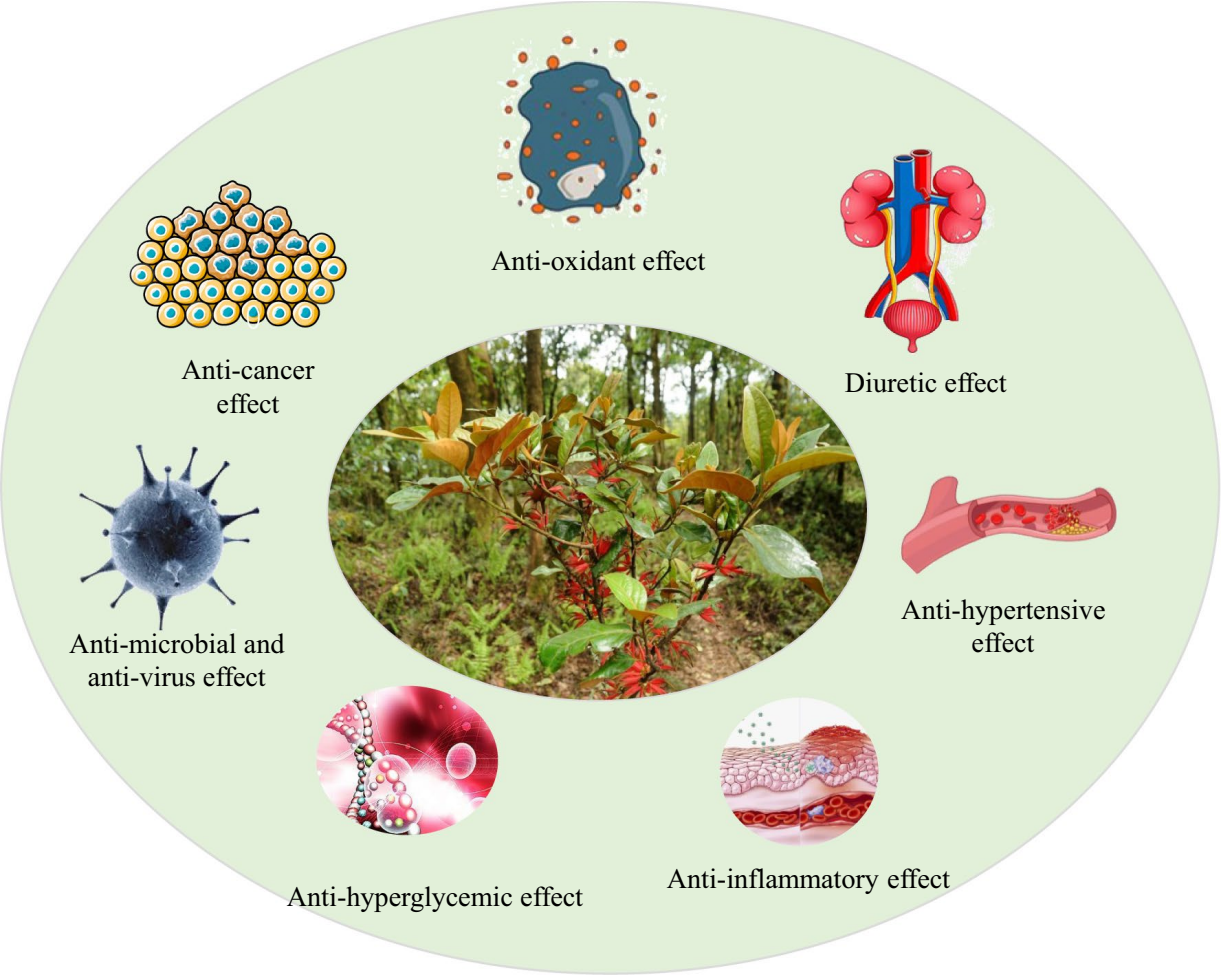
Summary of pharmacological effects of *Taxillus chinensis* (DC.) Danser

*T. chinensis* is a well-known Chinese medicine widely used to treat viral hepatitis, splenic asthenia, liver cancer, and renal toxicity [46]. Studies have shown that it has anti-inflammatory, antioxidant, anticancer, antimicrobial, antiviral, diuretic, antihypertensive, antihyperglycemic, and other properties (Fig. 4). The bioactivities are summarized in Table 4. *T. chinensis* “tonifying the liver and kidneys, strengthening muscles and bones” mainly involves biological processes such as cell proliferation, metabolic processes, biosynthesis processes, and inflammatory reactions by regulating thyroid hormone, osteoclast differentiation, HIF-1, TNF, NF-κB, and estrogen [47]. This process is closely related to the antioxidant, anti-inflammatory, antitumor, and cardiovascular protective activities of *T. chinens*is.

**Table 4:** Modern pharmacological studies of *Taxillus chinensis* (DC.) Danser

Bioactivities	Model	Extracts or compound	Method of administration	Dosage	Mechanism	References
Anti-inflammatory	RAW 264.7 macrophages	The whole dried plant/water/ethanol extracts	/	60 mg/mL	Inhibiting NO and TNF-α production	[52]
	RAW 264.7 macrophages	The whole plant/quercetin	/	2.5, 5, 10, 20 μg/ml	Inhibiting NO production and the protein expression of iNOS and COX-2 in a dose-dependent manner	[7]
	Xylene-induced ear edema mice	The whole plant/water extracts	Intragastric administration	0.5, 1.0 mg/kg b/w	Reducing the extent of ear edema	[55]
	Collagen-induced arthritis mice	The whole plant/quercetin	Intragastric administration	50, 100 mg/kg b/w	Inhibiting ankle mitochondrial dysfunction and mitochondrial oxidative stress	[4]
Antioxidant	DPPH radical	The whole dried plant/water/ethanol extracts	/	60 mg/mL	Exhibiting good free radical scavenging ability	[52]
	yeast model	The whole dried plant/water/ethanol extracts	/	60 mg/mL	Showing higher survival rates	[52]
	DPPH radical	Dried stem/the crude polysaccharide extract	/	10 mg/mL	Exhibiting significant scavenging ability, with scavenging capacities more than 400 µM ascorbate equiv/g	[68]
	*Saccharomyces cerevisiae* BY4734	Dried stem/the purified polysaccharide	/	10 mg/mL	TCP-2, TCP-3, UC-1 and UC-2 displaying significant antioxidant activity	[68]
Anticancer	Human colorectal cancer cell lines HCT116 and SW480	The whole plant/70% ethanol extracts	/	25, 50, 100 μg/ml	Inhibiting human colorectal cancer cells through GSK3β-dependent cyclin D1 degradation	[73]
	Human colon cancer HT-29 cells	Taxilli heba/water extracts	/	0, 0.5, 1, 2, 4, 8 g/mL	Inhibiting the proliferation, invasion, and migration of human colon cancer cells via the PI3K/AKT signaling pathway	[74]
	Human leukemia cell lines HL-60 and K562	The whole plant/extracts	/	50, 100, 200, 400 μg/ml	Inhibiting cell proliferation	[75]
	Human leukemia U937 cells	Quercetin	/	10, 20 μM	Promoting the reversible G2/M phase arrest of U937 cells	[80]
	Myeloid cell line P39	Quercetin	/	10, 50, 100 μM	Decreasing expression of BcL2, BcL-xL and McL-1 expression, increasing Bax expression	[81]
	Liver cancer cells (HuH-7 cells, HepG-2 cells)	Procyanidin B1	/	0, 5, 50, 500 μM	Inhibiting the Kv10.1 channel in a concentration-dependent manner	[82]
	Xenograft mice	Procyanidin B1	Subcutaneous injection	15 mg/kg b/w	Inhibiting the growth of the tumor	[82]
	MCF-7 and MDA-MB-231 breast cancer cell lines	Procyanidin C1	/	6.25 ~ 100 μg/mL	Inducing DNA damage, arresting the cell cycle, Decreasing Bcl-2 levels, and increasing the expression of BAX, caspase 3 and 9 in cells	[83]
	Human liver cancer cells Bel-7402 and human stomach cancer cells MGC-823	Lectins	/	6.25, 12.5, 25, 50, 100, 200 μg/mL	Inhibiting the growth of cells	[86]
Antimicrobial and anti-virus	Vero E6 cells	The dried stem, with leave of the plant/water–ethanol extracts	/	0, 25, 50, 100, 200 μg/mL	Inhibiting SARS-CoV 3CL protease activity	[87]
	HBsAg	The whole plant/10% water extract	/	20, 40, 80, 150, 300, 600, 1200, 2500 μg/50 *μ*L	Exhibiting good inhibitory effect on HbsAg	[88]
	DHBV-infected ducklings	Chlorogenic acid, caffeic acid, quinic acid	Intragastric administration	100 mg/kg b/w	Reducing the DHBV viremia	[89]
Diuretic	Anesthetized dogs	Avicularin	Intravenous injection	0.5 mg/kg b/w	Exhibiting diuresis	[95, 96]
	Rats	Avicularin	Intragastric administration or injection	34 mg/kg b/w	Exhibiting significant diuretic effect	[95, 96]
Anti-hypertensive	Spontaneously hypertensive rats	Taxilli herba	Intragastric administration	1.48, 5.90 g/kg b/w	Increasing SOD activity, increasing NO release in serum, and decreasing AngII and ET-1 levels in plasma	[102]
	Renal hypertensive rats	Quercetin	Intragastric administration	30 mg/kg b/w	Decreasing blood pressure, reducing the concentration of free calcium ions in renal artery smooth muscle cells	[103]
Anti-hyperglycemic	Hyperglycemia mice induced by streptozotocin	Total flavonoids	Intragastric administration	100, 200, 400 mg/kg b/w	Reducing the serum TC and TG levels and increase the serum HDL-G content of mice	[107]
	Diabetic mice induced by streptozotocin (STZ)	Total flavonoids	Intragastric administration	150, 300, 600 mg/kg b/w	Inhibiting lipid peroxidation in mice, protecting thymus atrophy and improving insulin resistance in diabetic mice	[108]
	HepG 2 cells	Taxilli herba	/	0.1, 0.01, 0.001 mg/mL	Promoting the metabolism of peripheral tissues and improving the sensitivity of liver cells to insulin	[109]
	Type 2 diabetes mellitus (T2DM) model mice	The dried plant/Ethanol extract	Intragastric administration	7.5, 15, 30 g/kg b/w	Improving high blood glucose level, liver and kidney complications, and protecting liver and kidney function	[110]

## Anti-inflammatory effect

*T. chinensis* can treat multiple inflammatory diseases, including osteoarthritis, chronic pelvic inflammation, chronic kidney disease, and rheumatoid arthritis [48–51]. The anti-inflammatory activity of *T. chinensis* is closely related to its traditional rheumatism-dispelling effect. Zhang et al. used RAW264.7 cells to test the anti-inflammatory activity of the water-alcohol extract of *T. chinensis*. They found that *T. chinensis* extract had significant anti-inflammatory activity by restraining the generation of NO (IC_50_ = 0.05 mg/mL) and TNF-α (IC_50_ = 0.14 mg/mL), and did not significantly affect cell viability [52]. TNF-α can activate the expression of nicotinamide adenine dinucleotide phosphate (NADPH) oxygenase, thereby promoting the synthesis of reactive oxygen species (ROS) by NADPH and further aggravating the inflammatory response of the body [53]. It can be speculated that *T. chinensis* extract exerts its anti-inflammatory effect mainly by reducing the oxidative stress state of the body. A lipopolysaccharide-induced cellular inflammation model was also used to evaluate the anti-inflammatory activity of *T. chinensis*. Liu et al. found that RAW264.7 macrophages cultured with quercetin (5, 10, and 20 μg/mL) and LPS (100 ng/mL) for 24 h exhibited no changes in cell viability. Compared with LPS alone, 10 and 20 μg/mL quercetin significantly inhibited NO generation. Meanwhile, quercetin dose-dependent inhibition of iNOS and COX-2 protein expression was observed in LPS-treated Raw264.7 cells [7]. The two aforementioned studies were conducted on RAW264.7 cells to study their anti-inflammatory activity. However, one cell model is not convincing enough and should be further validated in other cell lines, such as the J774A.1 and BMDM cell models. The anti-inflammatory mechanism of quercetin may be related to the reduction of inflammatory cytokines TNF-α, IL-1, and IL-6 by inhibiting LPS-induced activation and nuclear translocation of NF-κB and phosphorylation of extracellular signal-regulated kinase 1/2 (ERK1/2) and c-Jun N-terminal kinase [54]. In a subsequent study, the regulatory effect of *T. chinensis* on the above inflammatory factors was detected to reveal its possible mechanistic pathway, which represents significant progress in the study of the anti-inflammatory effects of *T. chinensis*.

In addition, the water extract of *T. chinensis* reduced the extent of ear edema in mice, which was induced by the proinflammatory agent xylene, and accelerated the dissipation of inflammation, and its therapeutic effect was equal to that of the positive control drug aspirin [55]. Shen et al. found that quercetin treatment significantly attenuated the inflammatory response and inhibited ankle mitochondrial dysfunction and mitochondrial oxidative stress in collagen-induced arthritis (CIA) mice [4]. The afore-mentioned studies have verified the in vivo anti-inflammatory effect and provided a scientific basis for the folk use of *T. chinensis* to treat various inflammatory diseases, especially rheumatoid arthritis; however, these studies are still in the initial stages, and clinical experimental data are lacking. Further research is required to validate these results.

## Antioxidant effect

Free radicals and ROS generated during human metabolism cause severe oxidative damage to large molecules, such as proteins, DNA, and lipids, which have been linked to heart disease, cancer, and atherosclerosis [56–59]. For example, ROS accumulation occurs during lung tumorigenesis. To maintain oxidative homeostasis, BACH1, Nrf2, and Maf transcription factors regulate the expression of antioxidant genes such as heme oxygenase-1 (HO-1) [60]. *T. chinensis* contains a variety of flavonoids. Flavonols are one of the most common classes of flavonoids in the diet and exhibit strong lipid peroxidation-inhibitory activity [61]. Some flavones prevent amplification and damage to other biomolecules, such as lipids, proteins, and DNA, by directly decreasing the levels of free radicals (hydroxyl, superoxide, and nitric oxide) and/or reactive species (such as hydrogen peroxide, peroxynitrite, and hypochlorous acid) in cells, thereby interfering with various oxidative stress-related events [62]. The antioxidant activity of *T. chinensis* was studied using the 2,2-diphenyl-1-pyridine hydrazide (DPPH) free-radical scavenging method and a yeast model. Ascorbate (vitamin C) was used as a positive control. Notably, *T. chinensis* showed significant activity in both the DPPH and yeast models. The scavenging capacity of the water extract was 152.4 μM ascorbate equiv/g, whereas that of the ethanol extract was 91.36 μM ascorbate equiv/g. Moreover, the correlation between total phenolic and flavonoid content and antioxidant properties was significant for aqueous and ethanolic extracts [52]. The antioxidant activity of the extracts prepared using different solvents was significantly different, primarily due to the polarity of the extraction agent. Moreover, the antioxidant capacities of *T. chinensis* differ significantly among different hosts. For example, the ethyl acetate and n-butanol extracts of *T. chinensis* from *Clausena lansium* (Lour.) Skeels showed good antioxidant activity, and 80% acetone extracts of *T. chinensis* fro*m Nerium indicum* showed the best antioxidant activity [63, 64].

Recently, the separation, characterization, and activity of polysaccharides in oriental medicine have become research hotspots due to their antitumor, antioxidative, antidiabetic, hypolipidemic, and immunoregulatory activities [65–67]. The antioxidant activity of *T. chinensis* polysaccharides was studied by DPPH and yeast assays for the first time. All polysaccharide fractions of *T. chinensis* had significant scavenging ability, ranging from 207 to 662 μM ascorbate equiv/g. Among them, TCP-2 and TCP-3 showed significant antioxidant activity, with scavenging capacities greater than 400 μM ascorbate equiv/g. *Saccharomyces cerevisiae* BY4743 was used to determine the antioxidant capacity of the purified polysaccharides in vivo using a high-throughput assay. They demonstrated the in vivo antioxidant activity of fractions, including TCP-2, TCP-3, UC-1, and UC-2. However, TCP-1 was toxic at high concentrations and exhibited no significant antioxidant activity in vivo. Therefore, no further studies on TCP-1 were conducted. These results suggest the potential of polysaccharides isolated from *T. chinensis* as natural antitumor agents [68]. In summary, phenols, flavonoids, and polysaccharides from *T. chinensis* have clear antioxidant effects. The presence of other antioxidant active components in *T. chinensis* must be investigated further. Owing to its potent antioxidant activity, *T. chinensis* is expected to become a natural antioxidant that can be used to prevent or treat diseases related to oxidative damage.

## Anticancer effect

Most traditional chemotherapy and radiotherapy focus on antiproliferative treatment [69]. The antiproliferative activity of herbal medicines provides hope for treating cancer, tumors, and other malignant diseases. *T. chinensis* contains various anticancer compounds such as rutin, quercetin, and phlorin [70–72]. Park et al. found that 70% ethanol extracts of *T. chinensis* (25–100 μg/mL) inhibited human colorectal cancer cell growth in a dose-dependent manner. The potential molecular mechanism involves the inhibition of the proliferation of human colorectal cancer cells through GSK3β-dependent degradation of cyclin D1 [73]. Feng et al. explored the effects of *T. chinensis* extract on the proliferation, invasion, and migration of human colon cancer HT-29 cells. The relative protein expression levels of p-PI3K and p-AKT were significantly decreased following treatment with *T. chinensis* extract, suggesting that *T. chinensis* extract inhibited proliferation, invasion, and migration through the PI3K/AKT signaling pathway [74]. These findings provide evidence for the development of *T. chinensis* as a chemopreventive or therapeutic drug for human colorectal cancer. However, cell invasion and migration are complex processes involving various signaling pathways, genes and cytokines. It is not rigorous to determine the mechanism of action solely based on the decreased relative expression levels of p-PI3K and p-AKT in cells, and more indicators need to be measured for verification. It is also necessary to obtain additional cell lines from colorectal cancer and xenografted mice to confirm their anticancer activity. The results showed that the extract of *T. chinensis* from *Casuarina equisetifolia* Forst had inhibitory effect on the proliferation of HL-60 and K562 cells, and the diethyl ether, ethyl acetate, and n-butanol fractions were identified as the active fractions of *T. chinensis* against leukemia cells in vitro for the first time. However, the active ingredient and its mechanism of action need to be further studied [75].

The components of *T. chinensis* have also shown anticancer activity. For example, several studies have confirmed that quercetin inhibits the growth and proliferation of different cell lines, such as lung, colon, breast, and prostate cancer cell lines [76–79]. In addition, quercetin promoted reversible G2/M phase arrest in U937 cells by decreasing cyclin D, E, E2F1, and E2F2, and increasing cyclin B [80]. Quercetin induced apoptosis and autophagy in P39 cells and increased cell blocks in the G1 phase. Quercetin treatment in vivo significantly reduced the tumor volume in P39 xenografts and verified the aforementioned in vitro findings [81]. These studies revealed the antitumor activity of quercetin, indicating that it is an attractive antitumor drug. Procyanidin B1 **(64)** was identified as an effective specific inhibitor that inhibits the Kv10.1 pathway in a concentration-dependent manner, thereby inhibiting HuH-7 and HepG2 cell migration and proliferation. In vivo, 15 mg/kg procyanidin B1 significantly inhibited tumor growth, with an inhibition rate of approximately 60.25%. Moreover, compared with cisplatin, procyanidin B1 has no side effects on normal metabolism in mice [82]. Therefore, procyanidin B1 is a promising anti-tumor drug. Procyanidin B1 is a tannin component in *T. chinensis*, but its content has not been quantitatively analyzed. Further research is required to determine whether it is an anti-tumor active component of *T. chinensis*. Procyanidin C1**(65)**, another tannin in *T. chinensis*, promotes apoptosis in MCF-7 and MDA-MB-231 breast cancer cells by inducing DNA damage, arresting the cell cycle, reducing Bcl-2 levels, and increasing the expression of BAX and caspases 3 and 9 [83]. Glycosides in different plants were highly cytotoxic to different cancer cell lines in early preclinical studies [84]. Studies on the anticancer activity of glycosides isolated from *T. chinensis* will be of great value; however, whether they will affect normal cells and verify their safety for normal cells remains to be determined. At present, research on the anti-tumor properties of *T. chinensis* mainly focuses on small molecular substances such as flavonoids, alkaloids, and other compounds, whereas research on macromolecular substances such as toxic proteins and lectins is limited. Studies have confirmed that lectins from *T. chinensis* have anti-tumor effects. The MTT method showed that the IC_50_ values of lectins in BEL-7402 liver cancer cells and MGC-823 gastric cancer cells were 24.2 μg/mL and 20.9 μg/mL, respectively. The inhibitory rate of lectin on liver and gastric cancer cells increases with increasing drug concentration within a certain range [85, 86]. In this experiment, the lectins from *T. chinensis* were believed to have a certain anti-tumor effect, but the lectins used were of low purity and needed to be further separated and purified to detect a single component. In addition, this study was carried out in a cell model, and the detailed mechanism of the anti-tumor effect of lectins from *T. chinensis* remains to be investigated further in vivo. In conclusion, flavonoids, tannins, and lectins in *T. chinensis* have anti-tumor effects, and their mechanism is mainly related to the degradation of cell cycle proteins and the inhibition of cell proliferation and migration through certain signaling pathways.

## Antimicrobial and antiviral effects

The *T. chinensis* extract has antiviral effects. Wen et al. evaluated the anti-SARS-CoV activity of *T. chinensis* in Vero E6 cells. The effective concentration of the *T. chinensis* extract for inhibiting the SARS coronavirus was between 25 and 200 μg/ml. Although the EC_50_ values of the *T. chinensis* extract (EC_50_ = 5.39 μg/ml) were approximately three times higher than those of valinomycin (VAL) (EC_50_ = 1.87 μg/ml), the significantly lower EC_50_ value (< 10 μg/ml) is interesting. Moreover, the potential toxicity of *T. chinensis* extract to Vero E6 cells was evaluated using the MTT assay with the CC_50_ value as an evaluation index. The CC_50_ value of the *T. chinensis* extract (CC_50_ > 500 μg/ml) was even higher than that of the positive control valinomycin (CC_50_ = 75.01 μg/ml), indicating that the *T. chinensis* extract did not interfere with cell growth and was generally safe for host cells [87]. Therefore, *T. chinensis* extract can inhibit the replication of SARS coronavirus with no or low toxicity to Vero E6 cells; therefore, it may be a useful candidate for future anti-SARS therapy. Moreover, a total of 150 μg/50 μL of *T. chinensis* aqueous extract treated with eight hemagglutination units of HBsAg for 4 h had an eightfold inhibitory effect on HbsAg. The results showed that *T. chinensis* was a natural inhibitor of the hepatitis B virus [88].

Various chemical constituents of *T. chinensis* exhibit antimicrobial and antiviral activities. In a duck hepatitis B virus (DHBV) model, chlorogenic acid (**61**) significantly reduced DHBV viremia in DHBV-infected ducklings, and its inhibitory efficacy was superior to that of lamivudine. Caffeic acid (**60**) also reduced DHBV viremia, but quinic acid (**50**) only slightly altered it. In a cellular model, all three compounds inhibited HBV DNA replication and HBsAg production [89]. The results showed that the three compounds can potentially inhibit the hepatitis B virus; however, further research is required to explore its mechanism of action. Studies have confirmed that toddacoumaquinone (**58**) is a potential inhibitor of the SARS coronavirus protease. It has a significant inhibitory effect on the main protease of SARS-CoV with a binding energy of − 7.8 kcal/mol [90]. Caffeic acid (**60**) is widely used to combat chronic infections induced by fungi, bacteria, and viruses [91, 92]. Current studies have mainly focused on in vitro models, and the antimicrobial and antiviral effects and their mechanisms in in vivo models must be evaluated further.

## Diuretic effect

Diuretics are one of the most commonly used drugs that increase urine flow and induce urinary sodium loss, and are used to treat hypertension, congestive heart failure, and edema [93]. However, the long-term use of diuretics has shown toxic effects on the human body. Contrastingly, the diuretic effect of traditional Chinese medicine is obvious, lasts longer, and does not cause electrolyte disorders; therefore, traditional Chinese medicines and Chinese medicine compound preparations with diuretic effects must be developed [94]. *T. chinensis* is mostly used to dispel wind dampness in clinical settings. Therefore, it has the potential to promote diuresis. Avicularin is a flavonoid isolated from Taxilli Herba, with confirmed diuretic effect. Intravenous injection of 0.5 mg/kg avicularin to anesthetized dogs can cause diuresis, and the diuretic effect becomes more significant as the dose increases. Gavage or injection of 34 mg/kg avicularin in rats also has a significant diuretic effect [95, 96]. Aldosterone, angiotensin II, antidiuretic hormone, and atrial natriuretic hormone are the four hormones in the renin-angiotensin system that are closely linked to fluid balance. Many diuretic experiments will measure these indices, so they can provide a reference for subsequently studying the diuretic mechanism of *T. chinensis*. Triterpenoids and polyphenols have been clinically proven to have diuretic effects and have certain reference values for research on the diuretic effect of *T. chinensis* [97, 98]. Polyphenols, including flavonoids and phenolic acids, are abundant in *T. chinensis* and are associated with antioxidant, anti-inflammatory, and anti-tumor effects. In the future, experimental studies of these polyphenols in *T. chinensis* should be conducted to determine whether they also have diuretic effects. Although only few studies have been conducted on the diuretic properties of *T. chinensis*, its potential is worth exploring.

## Antihypertensive effect

Millions of people have been estimated to die annually from cardiovascular diseases, particularly myocardial infarction and stroke [99]. Population growth and aging have led to a gradual increase in cardiovascular morbidity and mortality [100]. In clinical practice, *T. chinensis* is often used in combination to treat hypertension due to hyperactivity of the “liver yang type” with dizziness, tinnitus, anorexia, and dreaminess as the main symptoms, based on the traditional efficacy of “nourishing the liver and kidney, regulating blood vessels, and expelling wind” [101]. Recently, the pharmacological effects of *T. chinensis* in lowering blood pressure have been investigated. Zhang et al. investigated the antihypertensive activity of *T. chinensis* from six different hosts in spontaneously hypertensive rats (SHRs). Taxilli Herba from five hosts can reduce blood pressure; however, the effect varies depending on the host. The antihypertensive effects of these five hosts were as follows: *Salix babylonica* L., *Liquidambar formosana* Hance, *Morus alba* L., *Castanea mollissima* Bl., *and Camellia oleifera* Abel. The high-dose (5.9 g/kg body weight) and low-dose (1.48 g/kg body weight) Taxilli Herba extract from *Morus alba* L. showed a significant antihypertensive effect similar to that of captopril on SHRs, and the molecular mechanism may be related to increased SOD activity, increased serum NO release, and decreased plasma Ang II and ET-1 levels [102]. *T. chinensis* from *Camellia oleifera* Abel. and *Nerium oleander* L. showed obvious toxicity, which may cause damage to the body of SHR rats, resulting in no antihypertensive effect. Therefore, the relationship between blood pressure reduction and toxicity requires further study. Quercetin also had an obvious antihypertensive effect on renal hypertensive rats, and the effect was approximately equivalent to that of verapamil. Quercetin-treated rats had a decreased fluorescence density ratio of free calcium ions in the renal artery vascular ring smooth muscle cells, suggesting that the hypotensive mechanism is related to a reduction in the concentration of free calcium ions in renal artery smooth muscle cells. To further explore its in-depth antihypertensive mechanism, the whole-cell patch-clamp technique was used to observe the effect of quercetin on the relevant channel currents on the membrane of rat renal artery smooth muscle cells [103]. Furthermore, the antihypertensive effect mediated by quercetin is related to the regulation of renal arachidonic acid metabolism. In the future, it may be used as an independent and cost-effective complementary approach to treat hypertension and prevent organ damage caused by uncontrolled hypertension [104].

## Antihyperglycemic effect

Diabetes is a serious, chronic disease that develops when the pancreas does not produce enough insulin or when the body does not utilize insulin effectively [105]. The vast majority of patients with metabolic hyperglycemic diseases have type 2 diabetes [106]. Chen et al. applied total flavonoids (100, 200, and 400 mg/kg body weight) from *T. chinensis* to a streptozotocin (STZ)-induced hyperglycemia mouse model, and the results showed that 200 and 400 mg/kg total flavonoids reduced serum TC and TG levels and increased serum HDL-G levels in mice. The hypoglycemic effect of the total flavonoids (400 mg/kg body weight) was similar to that of metformin (60 mg/kg body weight), a known hypoglycemic compound. The results suggested that *T. chinensis* prevented and treated hyperglycemia and its complications, which provided a new idea for preventing and treating hyperglycemia using traditional Chinese medicine [107]. However, the mechanism of hypoglycemic activity is not fully understood, nor does it demonstrate the potential of *T. chinensis* as a new therapeutic drug. Meng et al. then studied the mechanism through which the total flavonoids from *T. chinensis* decrease blood sugar levels in STZ-induced diabetic mice. In vitro experiments revealed that total flavonoids inhibited the activity of α-glucosidase and α-amylase in a dose-dependent manner. The inhibition rate of total flavonoids on α-glucosidase was similar to that of acarbose, but the inhibition rate of α-amylase was much lower than that of acarbose, a well-known α-glucosidase inhibitor. Therefore, total flavonoids are strong α-glucosidase inhibitors and weaker α-amylase inhibitors. After four weeks of treatment with total flavonoids (150, 300, and 600 mg/kg body weight) in the STZ-induced hyperglycemia mouse model, the blood glucose content of mice in the high-dose group (600 mg/kg body weight) was close to that of mice in the metformin (60 mg/kg body weight) group. Total flavonoids significantly reduced blood sugar levels and enhanced glucose tolerance, indicating that insulin resistance in diabetic mice was enhanced. Simultaneously, after administration, the liver glycogen content, SOD activity, and thymus index increased, while MDA content decreased, suggesting that the hypoglycemic mechanism of total flavonoids in *T. chinensis* may be related to the inhibition of lipid peroxidation in mice, protection of thymus atrophy, and improvement of insulin resistance in diabetic mice [108]. The authors verified the hypoglycemic effect of *T. chinensis* using in vitro and in vivo experiments and clarified the potential of *T. chinensis* as an antidiabetic drug, pointing out directions for future experimental research. *T. chinensis* can also increase glucose consumption in human HepG2 cells by promoting the metabolism of peripheral tissues and improving the sensitivity of liver cells to insulin [109]. Luo et al. identified that the alcoholic extract of *T. chinensis* (30, 15, 7.5 g/kg body weight) treatment significantly ameliorated hyperglycemia and liver and kidney complications and protected liver and kidney function in an STZ-induced hyperglycemia mouse model. The mechanism may be related to the improvement of immune function, upregulation of anti-apoptotic factors, and downregulation of apoptosis-promoting and inflammation-promoting factor expression to maintain the functional state of hepatocytes and decrease the degree of renal cell injury [110]. Although this experiment showed the hypoglycemic effect and possible mechanism of action of *T. chinensis*, its dosage is relatively large, and high-dose medication may cause toxicity and side effects. It cannot reflect the real situation of a drug in the human body, and its clinical reference value is low. The antioxidant and antihyperglycemic effects of caffeic acid (**60**) have been demonstrated in STZ-induced mouse models of diabetes, and in the future, it could be considered a dietary supplement for diabetes [111].

## Other effects

In addition, *T. chinensis* was found to inhibit fatty acid synthase and decrease weight in rats in a preliminary study; the pharmacologically active ingredient was avicularin [8]. Therefore, avicularin may act as an anti-obesity and anti-dietary flavonoid. In future studies, avicularin should be assessed in vivo, and its mechanism of action should be investigated [112]. Recently, catechins (**12**) and their derivatives have attracted public attention because of their anti-tumor, antioxidative, and antimicrobial activities, in addition to easy availability, few toxic effects, and low cost [76, 113, 114]. Catechins have been shown to have nephroprotective activity and can prevent kidney damage caused by nephrotoxic drugs [115, 116]. In future studies, the safety and efficacy of catechins in treating acute kidney injury should be further explored to provide reference values for clinical treatment. Previously, *T. chinensis* was shown to reverse scopolamine-induced memory impairment in mice [117]. It is speculated to protect the nerves and enhance memory. However, no relevant research has been conducted in recent years. Therefore, further experiments are urgently required to clarify the mechanisms of action.

## Toxicology

To date, reports on *T. chinensis* toxicity are mainly related to liver and embryo toxicity. In the process of growth and development, the host transfers its characteristic components to *T. chinensis*, and if these components are toxic, they will affect the safety of *T. chinensis*, so the toxicity of *T. chinensis* is closely related to the host. Xia et al. investigated the acute toxicity and liver damage caused by water extracts of *T. chinensis* from six different hosts in zebrafish. The results showed that *T. chinensis* from *M. alba* was not hepatotoxic to zebrafish, whereas *T. trichocarpum*, *C. oleifera*, *S. babylonica*, *M. azedarach*, and *N. indicum* showed a certain degree of liver damage in zebrafish [118]. The dosage of drugs for the safe treatment of pregnant women and safe clinical use of *T. chinensis* have been investigated [119]. *T. chinensis* has certain genetic toxicity when the dosage is 20 g/kg body weight, and it has potential embryonic development toxicity when it reaches 40 g/kg body weight. In addition, no teratogenic effect was observed in rat embryos, but high-dose decoction showed embryo toxicity [120]. Since few studies have analyzed the toxicological data of *T. chinensis*, a thorough evaluation of the toxicological performance of this plant is needed.

## Conclusion and perspectives

**Fig. 5:**
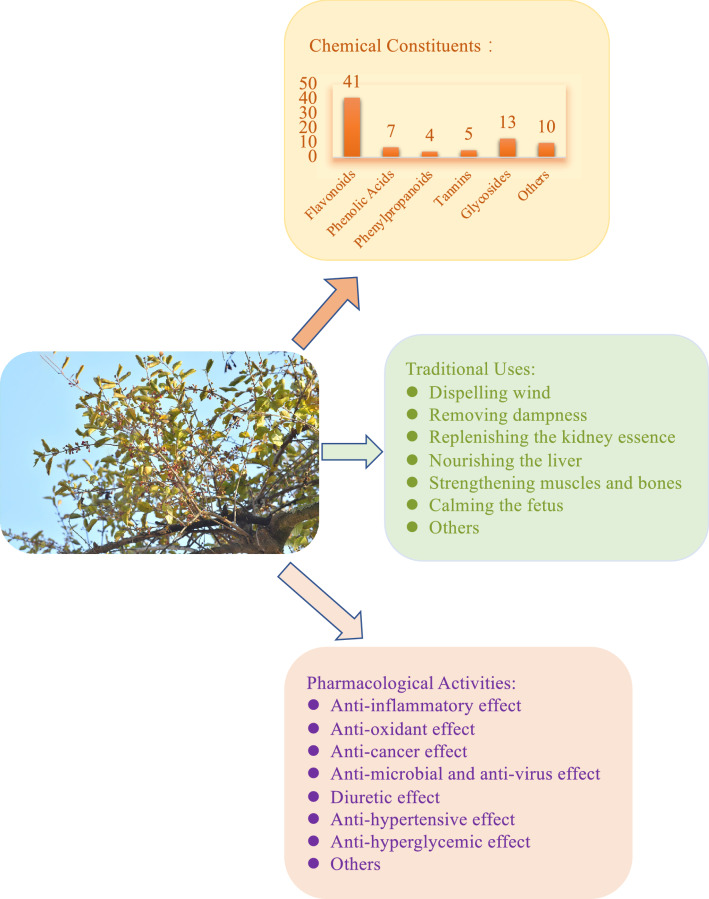
Traditional uses, chemical constituents, and pharmacological activities of *Taxillus chinensis* (DC.) Danser

As a traditional Chinese medicine, *T. chinensis* is abundantly available, contains various chemical components, and shows important pharmacological activities (Fig. 5). This paper summarizes the published research results on *T. chinensis* and reviews the botany, traditional uses, phytochemistry, pharmacology, and toxicity. The plant has been used in clinical practice since the reign of the Eastern Han dynasty. *T. chinensis* is traditionally used to treat various diseases, including joint swelling and pain, rheumatism, threatened abortion, stroke, and hypertension. The Duhuo Jisheng, Qisang Yigan, and Sangge Jiangzhi pills, among other preparations, have been widely used in traditional prescriptions and have shown good clinical effects. Phytochemical composition is an important factor in determining biological activity and traditional uses. Recently, researchers have isolated a variety of compounds from *T. chinensis*, among which quercetin has been widely studied, and it is the quality control index stipulated by the pharmacopoeia. *T. chinensis* has now been shown to possess various pharmacological activities, including anti-inflammatory, antioxidant, anticancer, antiviral, antimicrobial, antihypertensive, and antihyperglycemic activities.

To date, *T. chinensis* has been extensively investigated in various fields, and their phytochemical and pharmacological activities studied. However, further work on *T. chinensis* should focus on the following aspects. First, *T. chinensis* has a long history of application in China, and classic prescriptions with significant curative effects are available that are widely circulated and can treat various diseases. However, the relationship between traditional applications and modern pharmacological activities has not been sufficiently studied. Therefore, more experiments are required to elucidate the relationship between these compounds and to further discover their potential pharmacological activities. Second, approximately 110 compounds were identified in *T. chinensis*. However, most of the current studies are qualitative analysis of the compounds, and the exact content of each component in *T. chinensis* is not clear, which may be one of the future research focuses. Previous research has mainly focused on flavonoids; however, the pharmacological activities and targets of other components remain to be studied. Further exploration of the chemical components of *T. chinensis* is required to identify additional active components. What’s more, in practice, *T. chinensis* is often confused with mistletoe, resulting in market confusion and poor drug effect. In order to better control the quality of *T. chinensis*, it is necessary to establish a standardized fingerprint in the future. Third, although several pharmacological actions of *T. chinensis* have been reported, its exact active components and mechanisms are not clear, and pharmacological analysis is sometimes inconsistent with clinical application, resulting in confusion. Therefore, exploring the pharmacological action and mechanism of a single bioactive component will be the focus of future research. Finally, toxicological studies should be conducted to determine the safe dose of *T. chinensis* for clinical use, and clinical trials should be conducted to evaluate the efficacy of pharmacological actions in humans. Although this paper reviewed the botany, traditional application, phytochemistry, pharmacological activity and toxicity of *T. chinensis*, it lacked a summary of the pharmacokinetic research and clinical application. Clinical trials are essential to verify the safety and efficacy of drugs. In the future, clinical trials are necessary to develop more preparations related to *T. chinensis*. With the development of pharmaceutical chemistry and the improvement of human health level, the requirement of pharmacokinetics is increasingly high. In order to expand the market prospect of *T. chinensis*, future research can focus on the pharmacokinetics of the main active ingredients.

## Data Availability

All data generated or analyzed during this study are included in this published article [and its additional files].

## Abbreviations

*T. chinensis*: *Taxillus chinensis* (DC.) Danser
HIF-1: Hypoxia inducible factor-1
TNF: Tumor necrosis factor
NF-κB: Nuclear factor kappa-B
BACH1: Basic leucine zipper transcription factor 1
MAF: Minor allele frequency
Nrf2: Nuclear factor erythroid-2 related factor 2
HO-1: Heme oxygenase-1
DPPH: 1,1-Diphenyl-2-picrylhydrazyl
Bcl-2: B-cell lymphoma-2
MTT: 3-(4,5-Dimethyl-2-thiazolyl)-2,5-diphenyl tetrazolium bromide
DHBV: Duck hepatitis B virus
SHRs: Spontaneously hypertensive rats
STZ: Streptozotocin
iNOS: Inducible nitric oxide synthase
CC_50_: The half-maximal cytotoxic concentration
EC_50_: Concentration for 50% of maximal effect
IC_50_: The half maximal inhibitory concentration
LPS: Lipopolysaccharide
COX-2: Cyclooxygenase 2
p-PI3K: Phosphorylated phosphatidylinositol 3 kinase
p-AKT: Phosphorylated AKT
